# Epitaxy of ultrathin Fe_3_O_4_ films on SrTiO_3_(001): influence of growth parameters on the formation of coexisting (111)- and (001)-oriented phases

**DOI:** 10.1107/S1600576726003316

**Published:** 2026-05-20

**Authors:** Andreas Alexander, Kevin Ruwisch, Jannis Thien, Alexander Weissbach, Tobias Pollenske, Bin Zhang, Fabiola Schulte, Olof Gutowski, Alina Vlad, Alessandro Coati, Joachim Wollschläger

**Affiliations:** a Department of Physics Osnabrück University Barbarastraße 7 49076 Osnabrück Germany; b Deutsches Elektronen-Synchrotron DESY Notkestraße 85 22607 Hamburg Germany; c Synchrotron SOLEIL L'Orme des Merisiers Départementale 128 91190 Saint-Aubin France; Montanuniversität Leoben, Austria

**Keywords:** magnetite, epitaxy, thin films, grazing-incidence X-ray diffraction, GIXRD, high-energy X-ray diffraction, HEXRD

## Abstract

Ultrathin Fe_3_O_4_ films were grown on SrTiO_3_(001) substrates under systematically varied growth conditions (deposition rate and temperature, and film thickness) in order to determine the influence of these parameters on the formation of the coexisting (111) and (001) orientations of Fe_3_O_4_. Structural characterization was performed using grazing-incidence X-ray diffraction and high-energy X-ray diffraction, including mapping of the reciprocal space in plane and out of plane to obtain detailed information about the structure of the films in the lateral and vertical directions. Despite an expected cube-on-cube growth, an intermediate layer of (111)-oriented Fe_3_O_4_ with hexagonal surface symmetry formed in all samples examined. This intermediate phase occurs in the form of 3D islands during the initial growth phase and persists even after growth has transformed to preferential formation of the (001) orientation. The fraction of the (111) phase, on the other hand, depends heavily on the kinetic conditions during growth. Its formation is favoured by low deposition rates and, above all, high deposition temperatures. These results support earlier observations and provide new insights into the structural development of Fe_3_O_4_ films on SrTiO_3_(001) in the early stages.

## Abstract 2

Ultrathin Fe_3_O_4_ films grown on SrTiO_3_(001) were systematically studied with respect to film growth parameters to clarify the coexistence of (111) and (001) orientations. X-ray diffraction analyses reveal the formation of an intermediate (111)-oriented phase during the initial growth stage, which persists even after preferential (001) growth is established.

## 1. Introduction

Magnetite (Fe_3_O_4_) is of great interest as a candidate for applications in the fields of spintronics (Hoffmann & Bader, 2015 ▸; Hamie *et al.*, 2012 ▸; Seneor *et al.*, 1999 ▸; Kado, 2008 ▸; Wada *et al.*, 2010 ▸; Moussy, 2013 ▸) and spin caloritronics (Ramos *et al.*, 2016 ▸, 2013 ▸; Uchida *et al.*, 2016 ▸) due to a predicted 100% spin polarization at the Fermi level (Zhang & Satpathy, 1991 ▸). However, this promise has not met expectations, which might be due to interface effects (van der Zaag *et al.*, 2000 ▸; Jordan *et al.*, 2006 ▸; Marnitz *et al.*, 2015 ▸; Hu & Suzuki, 2002 ▸; Kado, 2008 ▸). For this reason, detailed knowledge of the growth of ultrathin magnetite films is necessary to control interface and surface properties required for high-quality spintronic devices (Moussy, 2013 ▸; Jordan *et al.*, 2006 ▸).

For the growth of magnetite ultrathin films, various substrates have been used, with MgO(001) being the most widespread. Since its lattice constant results in a small mismatch of 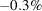 between half the lattice constant of Fe_3_O_4_ and MgO, ordered pseudomorphic growth is given (Bertram *et al.*, 2013 ▸, 2012 ▸, 2011 ▸; Wollschläger, 2018 ▸; van der Zaag *et al.*, 2000 ▸; Chang *et al.*, 2016 ▸; Celotto *et al.*, 2003 ▸; Margulies *et al.*, 1997 ▸; Tobin *et al.*, 2007 ▸). However, growth and annealing of Fe_3_O_4_ on MgO(001) substrates is limited in temperature, since above 250°C Mg^2+^ interdiffuses into the magnetite film (Wollschläger, 2018 ▸; Kim-Ngan *et al.*, 2009 ▸).

It has been demonstrated that interdiffusion of ferrite and SrTiO_3_(001) substrates at elevated temperatures is prohibited (Rodewald *et al.*, 2019 ▸; Thien *et al.*, 2020 ▸). Therefore, despite a large lattice mismatch of 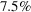 between the SrTiO_3_ substrate and half the lattice constant of Fe_3_O_4_, magnetite films can also be grown on it, even at elevated temperatures (Kuschel *et al.*, 2016 ▸). Furthermore, due to the large lattice mismatch, the use of SrTiO_3_(001) as a substrate offers the possibility of studying strain effects, which can be used to modify the electronic and magnetic properties of Fe_3_O_4_ (Kuschel *et al.*, 2016 ▸; Monti *et al.*, 2013 ▸; Kale *et al.*, 2001 ▸; Rubio-Zuazo *et al.*, 2015 ▸; Liu *et al.*, 2017 ▸; Chen *et al.*, 2008 ▸).

For the growth of Fe_3_O_4_ on SrTiO_3_(001), cube-on-cube growth is expected and is also frequently observed (Kuschel *et al.*, 2017 ▸; Pohlmann *et al.*, 2022 ▸; Alexander *et al.*, 2025 ▸; Wollschläger, 2018 ▸). However, for high temperatures, *i.e.T* ≥ 700°C, Fe_3_O_4_ has been reported to grow in its (111) orientation with hexagonal surface symmetry (Takahashi *et al.*, 2012 ▸, 2014 ▸; Leung *et al.*, 2008 ▸). This effect is attributed to both the large lattice mismatch (7.5%) and the anisotropy of the surface energy of the spinel structure of Fe_3_O_4_, which leads to competition between (111) and (001) growth orientations (Mishra & Thomas, 1977 ▸; Yu *et al.*, 2012 ▸; Santos-Carballal *et al.*, 2014 ▸). In recent work, using surface-sensitive spot-profile-analysis low-energy electron diffraction (LEED) measurements, the (111) orientation of Fe_3_O_4_ on SrTiO_3_(001) was observed at even lower temperatures (250°C) as an initial interface structure before it continues to grow in the (001) orien­tation with cubic surface structure (Alexander *et al.*, 2025 ▸).

In this work we analyse the conditions under which the (111) orientation of Fe_3_O_4_ is preferentially formed and their influence on the growth behaviour. In particular, the aim is to find out whether the (111) orientation is only a temporary intermediate phase that is transformed into the (001) orientation in the further growth of the thin film or whether this phase persists and coexists with the later (001) orientation.

For this reason, in this work, ultrathin magnetite films have been grown on SrTiO_3_(001) by reactive molecular beam epitaxy (RMBE) and analysed *in situ* by means of synchrotron-radiation-based (grazing-incidence) X-ray diffraction (GIXRD) and high-energy X-ray diffraction (HEXRD), providing detailed structural information of the surface, interface and film structure. By varying the growth parameters, their influence on film growth and the formation of the (111) orientation of Fe_3_O_4_ was studied.

## 2. Experimental details

The magnetite thin films were prepared and *in situ* characterized at the SixS beamline of Synchrotron SOLEIL (Saint-Aubin, France). Here, the diffractometer is coupled to three ultra-high vacuum chambers with a base pressure of 2 × 10^−10^ mbar, enabling (grazing-incidence) X-ray diffraction experiments directly after film growth without breaking the vacuum (Dawiec *et al.*, 2016 ▸).

Before deposition, the SrTiO_3_(001) substrates were cleaned by annealing for 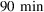 at 420°C in a molecular oxygen atmosphere of 5 × 10^−5^ mbar in order to remove adsorbates.

**Figure 1:**
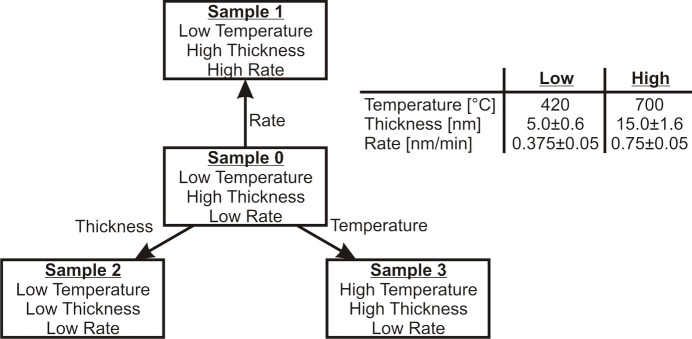
Scheme of studied samples including experimental parameters such as growth temperature, film thickness and deposition rate. First, sample 0 (S0) was analysed as a reference. Subsequently, only one parameter was varied at a time in order to analyse its influence on the structure.

The films were grown by RMBE, *i.e.* thermal evaporation from a pure Fe target in a molecular oxygen atmosphere of 5 × 10^−6^ mbar. During deposition of the first film, the substrate temperature was kept at 420°C and a constant deposition rate was controlled by the flux of the evaporator. This procedure was used to calibrate the deposition rate as recorded by a quartz crystal microbalance prior to the experiments. Afterwards, further samples were prepared by varying one growth parameter at a time, *i.e.* deposition rate, film thickness and temperature (*cf.* Fig. 1 ▸). Thus, the influence on the growth behaviour of each parameter can be analysed separately.

For the following diffraction experiments, a photon energy of 16 keV was used and the data were collected by an XPAD S140 two-dimensional detector. In order to obtain information on the out-of-plane structure of the films, X-ray diffraction (XRD) was performed using the θ–2θ geometry. Subsequently, using a grazing-incidence geometry (GIXRD) with a fixed glancing angle of 0.2°, scans along several crystal truncation rods (CTRs) attributed to Fe_3_O_4_(001) and Fe_3_O_4_(111) were performed. In the following, the two different Fe_3_O_4_ orientations, (001) and (111), are labelled ‘cub’ and ‘hex’, respectively, referring to the square and hexagonal surface structure, respectively. Moreover, two-dimensional in-plane mappings were recorded.

After transport under ambient conditions, films were additionally studied by means of HEXRD at the P07 beamline (EH2) of PETRA III at DESY. For the diffraction experiment, a photon energy of 73.3 keV and a glancing angle of θ = 0.03° were used. The data were collected by a Varex XRD 4343 CT 2D area detector. During the measurement, the samples were rotated between ±90° with a rotation speed of 0.5° s^−1^ to collect the diffraction signal from the entire sample. Afterwards, the detector images of these scans were summed in order to obtain a full reciprocal-space map of the entire film.

## 3. Results

### 3.1. Reference sample S0

**Figure 2:**
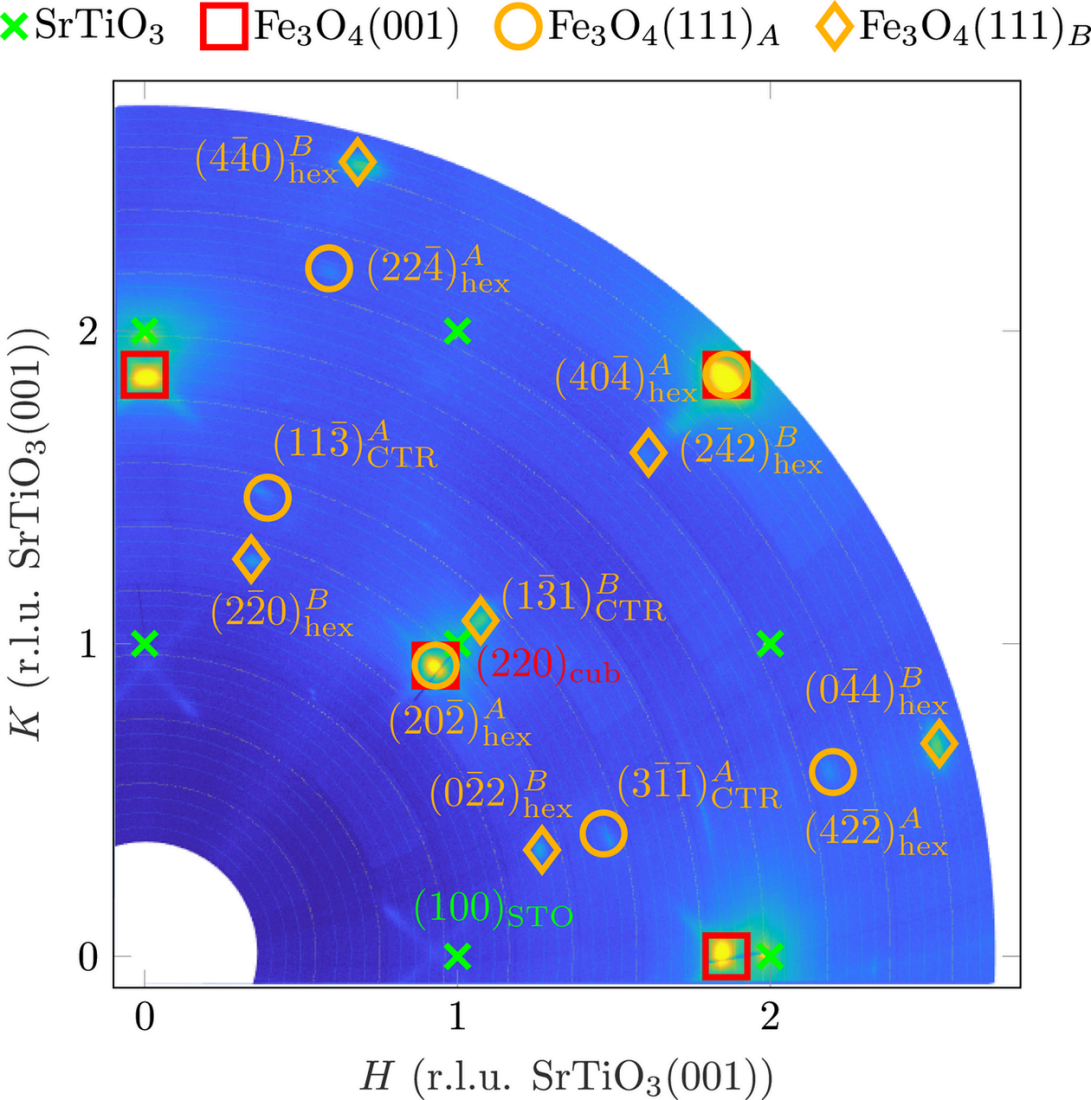
Section of an in-plane reciprocal-space map of reference sample for summation of intensities running the scaled out-of-plane scattering-vector magnitude *L* between 0.02 and 0.1 [r.l.u. SrTiO_3_(001)]. For reasons of clarity, only reflections/CTRs that can be detected are included.

Fig. 2 ▸ shows an in-plane reciprocal-space map for low *L*values {0.02 < *L* < 0.1 [r.l.u. (reciprocal-lattice units) SrTiO_3_(001)]}. Besides the expected reflections of the SrTiO_3_ substrate (green crosses) and reflections of the cubic (001) orientation of Fe_3_O_4_ (red squares), reflections with a 12-fold symmetry can be found. These are reflections of the hexagonal surface structure of Fe_3_O_4_(111), which is present in two domains, *A* (yellow circles) and *B* (yellow diamonds), rotated by 90°, as well as the respective mirror domains 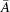 and 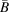. The latter two domains are not found in Fig. 2 ▸ as their in-plane reflections fully overlap with reflections from *A* and *B*. However, the mirror domains can be distinguished using higher out-of-plane scattering vectors (higher values of *L*).

**Figure 3:**
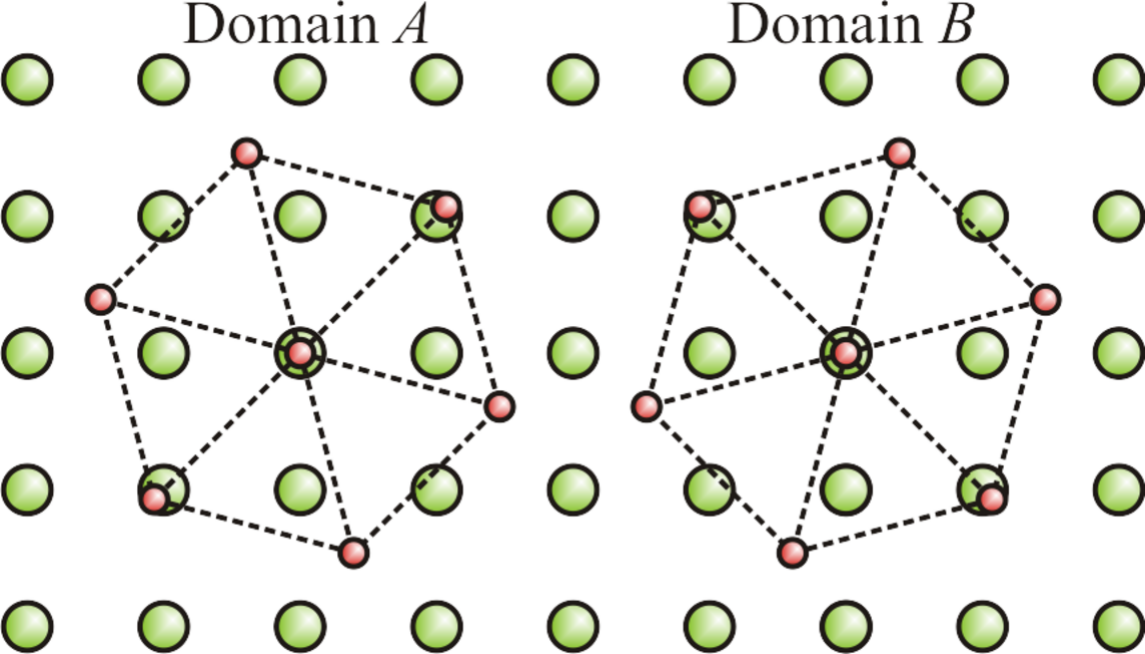
Schematic model for the in-plane orientation of Fe_3_O_4_(111) grown on SrTiO_3_(001). The larger green circles denote the Sr^2+^ cations and the smaller red circles represent the Fe^3+^ cations of the Fe_3_O_4_(111) unit cell. For simplicity, Ti and O atoms are not shown and the surface unit cell of Fe_3_O_4_(111) is used. The two possible domains, *A* and *B*, rotated by 90°, are shown. [Adapted from Leung *et al.* (2008 ▸).]

Since 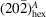 coincides with (220)_cub_ [close to (110)_STO_], it is evident that the orientation of Fe_3_O_4_(111) with respect to the substrate is
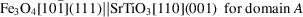
and
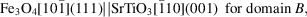
and analogously for their mirror domains 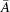 and 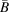, respectively. A graphical representation of the orientation of Fe_3_O_4_(111) on the substrate is shown in Fig. 3 ▸. The 12-fold symmetry and the positions of the in-plane Fe_3_O_4_(111) reflections show that the Fe_3_O_4_(111) layer is (almost) fully relaxed. This result contradicts the model proposed by Takahashi *et al.* (2012 ▸), which suggests that the Fe_3_O_4_(111) film adapts to the lattice of the SrTiO_3_(001) substrate in the sense of a superstructure. This would lead to distorted diffraction patterns with a sixfold instead of a 12-fold symmetry.

The cross-shaped weak intensities around the Fe_3_O_4_(001) reflections indicate facets with an in-plane component in the 〈110〉 directions.

Since only the square surface structure of the Fe_3_O_4_(001) can be seen with surface-sensitive electron diffraction (LEED), here it can be concluded from the presence of the Fe_3_O_4_(111) reflections that the (111) orientation is not a temporary intermediate phase that transforms into the (001). Rather, a permanent Fe_3_O_4_(111) interlayer is stabilized at the interface that continues to coexist with the Fe_3_O_4_(001) film.

**Figure 4:**
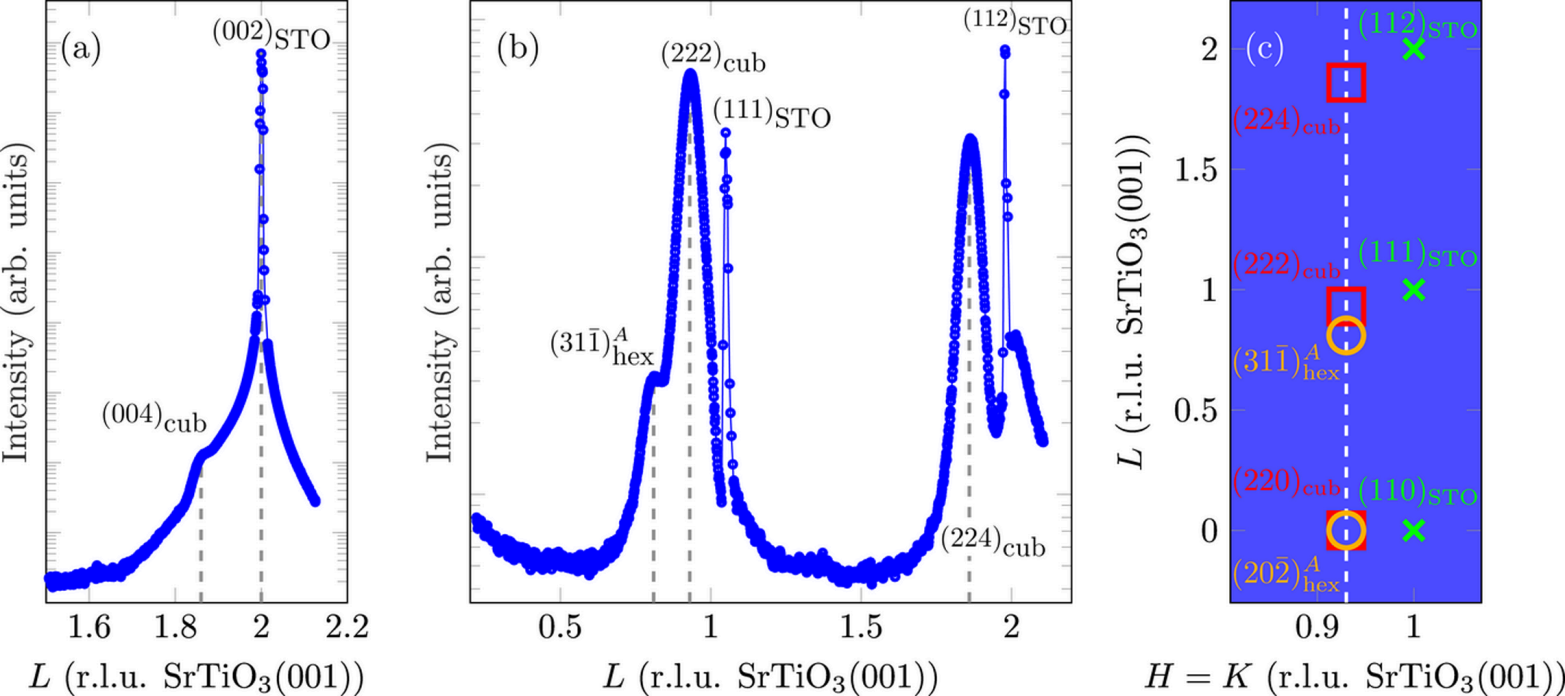
(*a*) XRD scan along the (00)-CTR of the reference sample. Since nothing can be seen in the range *L* < 1.5 except for the substrate reflection, this is not shown. (*b*) *L* scan along the 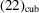-CTR of Fe_3_O_4_(001) of the reference sample (*H* ≈ *K* ≈ 0.93, *cf.* Fig. 2 ▸). Here, the sharp outliers at *L* = 1 and *L* = 2 are due to diffuse scattering close to very intense substrate Bragg peaks being close to the measured Fe_3_O_4_ CTR. (*c*) Schematic sketch of the reflections expected on and near the 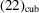-CTR of completely relaxed Fe_3_O_4_(001) and Fe_3_O_4_(111).

Fig. 4 ▸(*a*) presents a (00*L*) XRD scan performed at the reference sample. Besides the expected reflections of the substrate, the (004)_cub_ reflection can be seen due to (001)-oriented (majority) fractions of the Fe_3_O_4_ film. Near this Bragg peak, no Laue fringes are observed, pointing to an inhomogeneous crystalline order of Fe_3_O_4_(001).

Furthermore, the (222)_hex_ reflection, expected at *L* = 1.62, is not present. Although the intensity of Fe_3_O_4_(222) is weak compared with Fe_3_O_4_(004) due to its structure factor, the complete absence of the reflection indicates that most of the Fe_3_O_4_ film grows with the (001) orientation.

The full width at half-maximum (FWHM) of (004)_cub_ is used to determine the crystalline film thickness *d*_cryst_ according to the Scherrer formula (Scherrer, 1918 ▸):
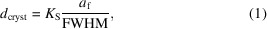
with *K*_S_ = 0.89 and *a*_f_ being the bulk lattice constant of Fe_3_O_4_. This results in a crystalline film thickness *d*_cryst_ = 14.0 (2) nm. A comparison with the deposited film thickness shows that *d*_cryst_ is only slightly lower, meaning that most of the layer is ordered crystalline.

Fig. 4 ▸(*b*) shows the diffractogram recorded for the 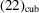-CTR of Fe_3_O_4_(001). Fe_3_O_4_ reflections due to both Fe_3_O_4_(001) and Fe_3_O_4_(111) can lie on this CTR [*cf.* Fig. 4 ▸(*c*)]. Besides the two Fe_3_O_4_-related reflections (222)_cub_ and (224)_cub_ at *L* = 0.93 and *L* = 1.86, respectively, an additional reflection is present at *L* = 0.81. This reflection is attributed to 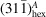 of Fe_3_O_4_(111).

The intensities of (222)_cub_ and 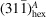 are used to determine the quantity of (111)-orientated Fe_3_O_4_. For this, the structure factors *F*_*HKL*_ of the respective reflections must be taken into account. Structure factors were calculated from the spinel structure using atomic form factors from Brown *et al.* (2006 ▸). Consequently, the normalized intensity yield *Y*_hex/cub_, *i.e.* the fraction of Fe_3_O_4_ with (111) orientation, can be calculated as
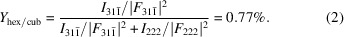
Only the Fe_3_O_4_(111) reflections of one domain and its mirror domain (here, *A* and 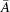) are apparent at this position. Assuming a statistically uniform distribution of the domains, the overall fraction of Fe_3_O_4_ with (111) orientation is twice as high, *i.e.*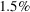.

Taking this result and the respective structure factors into account, it is obvious that the (222)_hex_ reflection, which would be expected at approximately *L* = 1.62 on the (00*L*) CTR, is not visible in the XRD measurement [*cf.* Fig. 4 ▸(*a*)], as its intensity is only 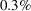 of that of (004)_cub_ and it therefore vanishes into the background. The same applies to (111)_hex_, expected at *L* = 0.81.

From the vertical difference between the two reflections (222)_cub_ and (224)_cub_, a vertical lattice constant of *a*_⊥_ = 839.2 (10) pm can be determined. Within the experimental uncertainty, this is in good agreement with the value of 839.6 pm for bulk magnetite (Cornell & Schwertmann, 2003 ▸), demonstrating again complete relaxation of the Fe_3_O_4_(001) film in the vertical direction. A detailed discussion will be presented later.

**Figure 5:**
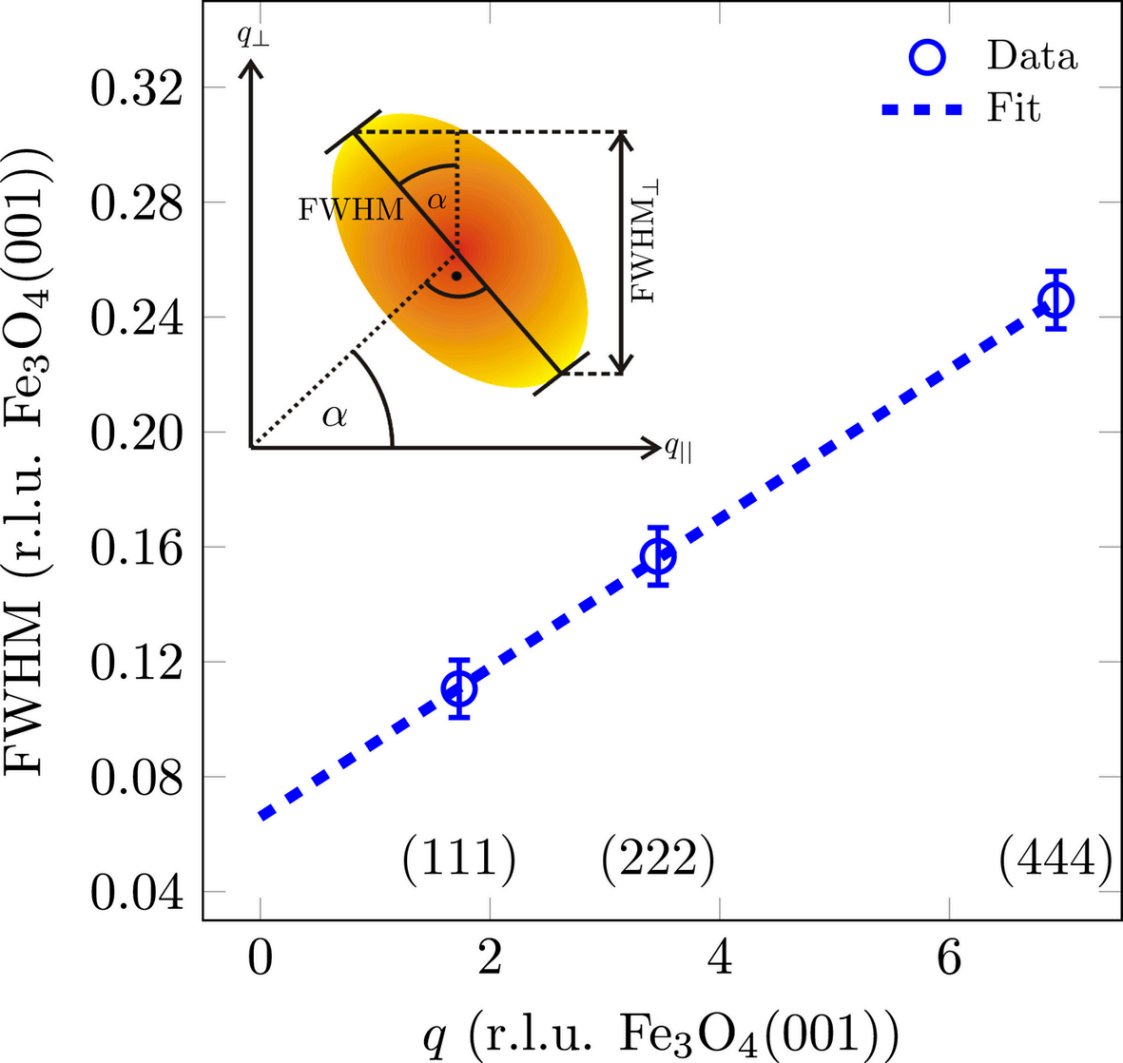
FWHM analysis of (*LLL*) reflections of (001)-oriented Fe_3_O_4_ of the reference sample as a function of the scattering-vector magnitude *q*. The FWHM of the peaks has been fitted with a linear function. The inset shows the projection of the effective FWHM onto the measured vertical FWHM_⊥_.

The FWHM analysis of the reflections shows that for Fe_3_O_4_(001) there is a broadening of the FWHM with increasing scattering-vector magnitude *q*, which is nearly linear (*cf.* Fig. 5 ▸). This broadening with increasing scattering vector indicates mosaics, *i.e.* parts of the surface that are slightly tilted with respect to the SrTiO_3_(001) surface. In this case, the broadening of the spot profiles is related to the spread of the tilted mosaics, *i.e.* the sharpness of the distribution of mosaic angles, which is highly dependent on the lattice mismatch (Wollschläger *et al.*, 2001 ▸). The slope of the linear incline corresponds to the mosaic spread Δσ, while the FWHM for the extrapolation 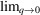 is caused by finite size effects resulting from the size of the mosaics. These mosaics can be attributed to the formation of misfit dislocations at the interface for strain release caused by the lattice mismatch between Fe_3_O_4_(001) and SrTiO_3_(001) as observed in different material systems with high lattice mismatch (Goldbach & Wollschläger, 2022 ▸; Wollschläger *et al.*, 2001 ▸; Dynna *et al.*, 1996 ▸; Vassent *et al.*, 1996 ▸). Since the broadening of the reflections is perpendicular to the origin or to the scattering vector **q** but the *L* scan only measures the vertical component of the FWHM_⊥_, a corresponding correction must be made for each measured FWHM_⊥_ in order to calculate the projection of the FWHM (*cf.* inset in Fig. 5 ▸).

A fitting of the FWHM with a linear function (*cf.* Fig. 5 ▸) results in a mosaic spread of Δσ = 1.82 (4)° and a constant offset of 0.066 (r.l.u. Fe_3_O_4_) that corresponds to a lateral mosaic size of *d*_mosaic_ = 11.3 (2) nm with respect to the [111] direction.

### 3.2. Influence of deposition rate

The first variation in film growth parameters was a higher deposition rate, achieved by increasing the material flux of the evaporator. Therefore, the deposition rate was increased from 0.375 to 0.750 nm min^−1^ while keeping the deposition temperature and final film thickness constant. For the latter, the deposition time was halved.

**Figure 6:**
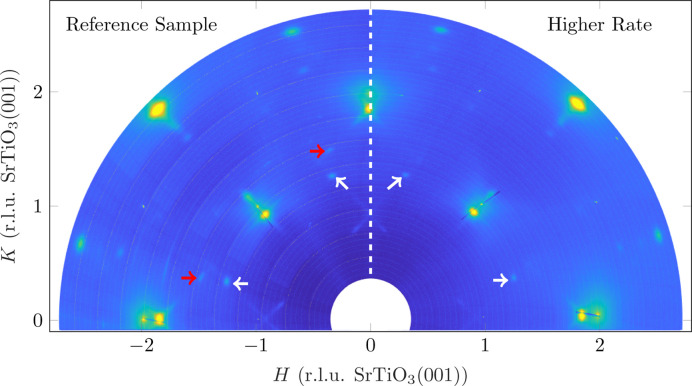
Comparison of the in-plane reciprocal-space maps of the reference sample (left) and the sample with higher deposition rate (right). The *L* value is a summation between 0.02 and 0.1 [r.l.u. SrTiO_3_(001)] (*cf.* Fig. 2 ▸). The white arrows denote in-plane reflections of the (111) orientation, while the red arrows denote CTRs of the (111) orientation without in-plane reflections.

Qualitatively, no significant difference can be seen in the in-plane maps with a change in the deposition rate (*cf.* Fig. 6 ▸). The reflections of the (111) orientation (white arrows) are slightly more pronounced at a lower deposition rate, especially the non-integer CTRs without in-plane reflections (red arrows). In addition, the crosses caused by facets on the Fe_3_O_4_(001) reflections are slightly more pronounced, which indicates that the proportion of facets has increased, *i.e.* larger facets have been formed or more facets have been formed.

**Figure 7:**
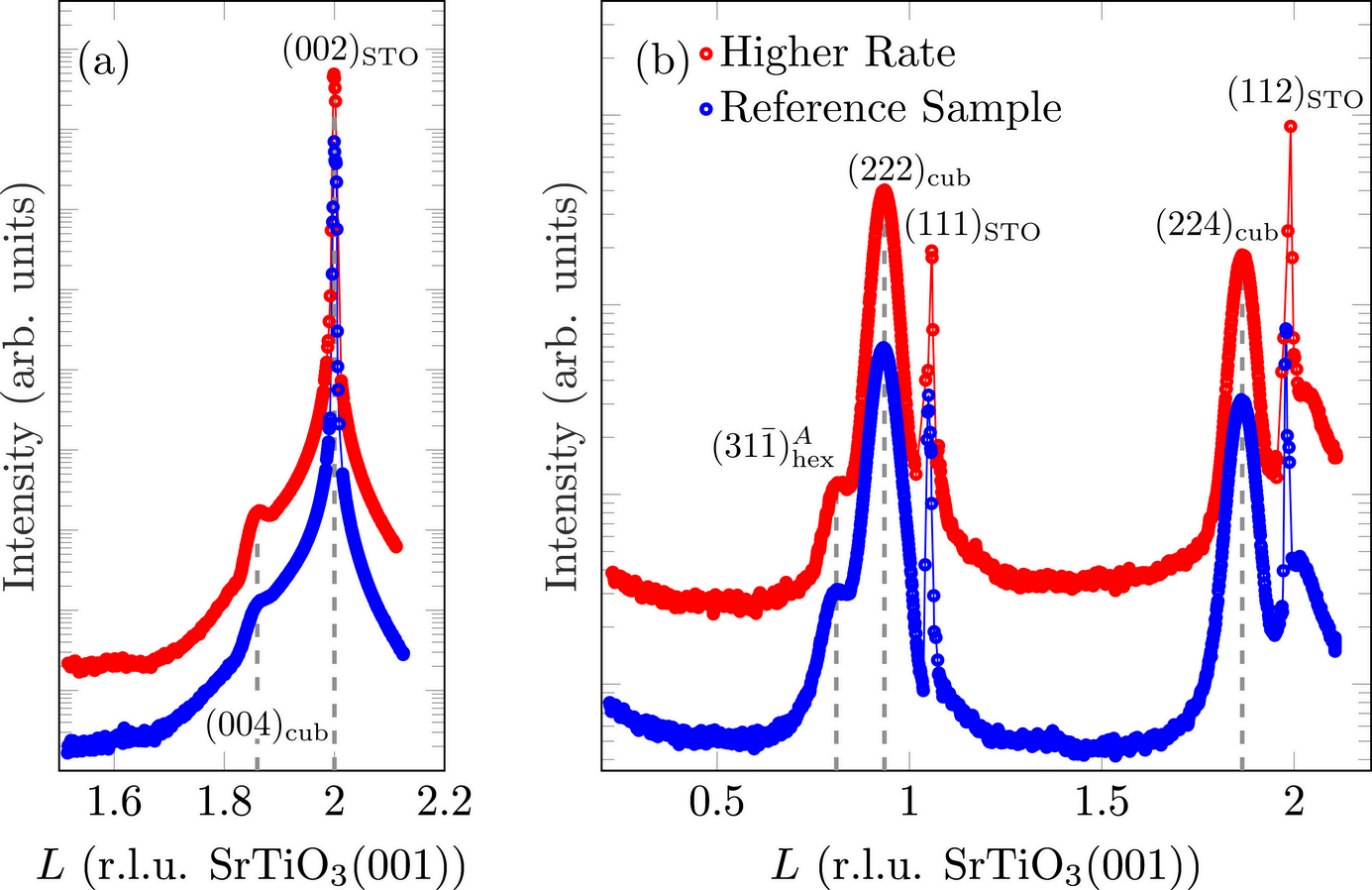
(*a*) Comparison of XRD scans along the (00)-CTR for different deposition rates. (*b*) Comparison of *L* scans along the 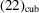-CTR of Fe_3_O_4_(001) for different deposition rates. Here, the sharp outliers at *L* = 1 and *L* = 2 are due to diffuse scattering close to very intense substrate Bragg peaks being close to the measured CTRs.

In the XRD measurements on the (00*L*) rod with a higher deposition rate, there is also no significant difference from the reference sample [*cf.* Fig. 7 ▸(*a*)]. The (004)_cub_ reflection has a slightly higher intensity, so that it is more apparent.

The FWHM of (004)_cub_ gives a crystalline layer thickness of *d*_cryst_ = 12.9 (2) nm according to equation (1). Despite the initial deposited volume of Fe_3_O_4_ being the same, this is slightly lower than for the reference sample, which could be explained by the fact that the layer with the lower growth rate can arrange its structure better.

Equally on the (22)_cub_-CTR, no significant qualitative difference can be seen between the two samples [*cf.* Fig. 7 ▸(*b*)]. The reflection 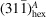 belonging to the (111) orientation of Fe_3_O_4_ can also be seen for both preparations. Using the intensity ratio of (222)_cub_ and 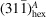 according to equation (2), the fraction of (111)-orientated Fe_3_O_4_ (both *A* and 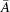 domains) in the sample with the higher deposition rate is 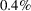. This is a decrease by half compared with the reference sample, suggesting that a lower deposition rate favours the formation of the (111) orientation, and supports the qualitative observations from the comparison of the in-plane maps for the two preparations (*cf.* Fig. 6 ▸).

**Figure 8:**
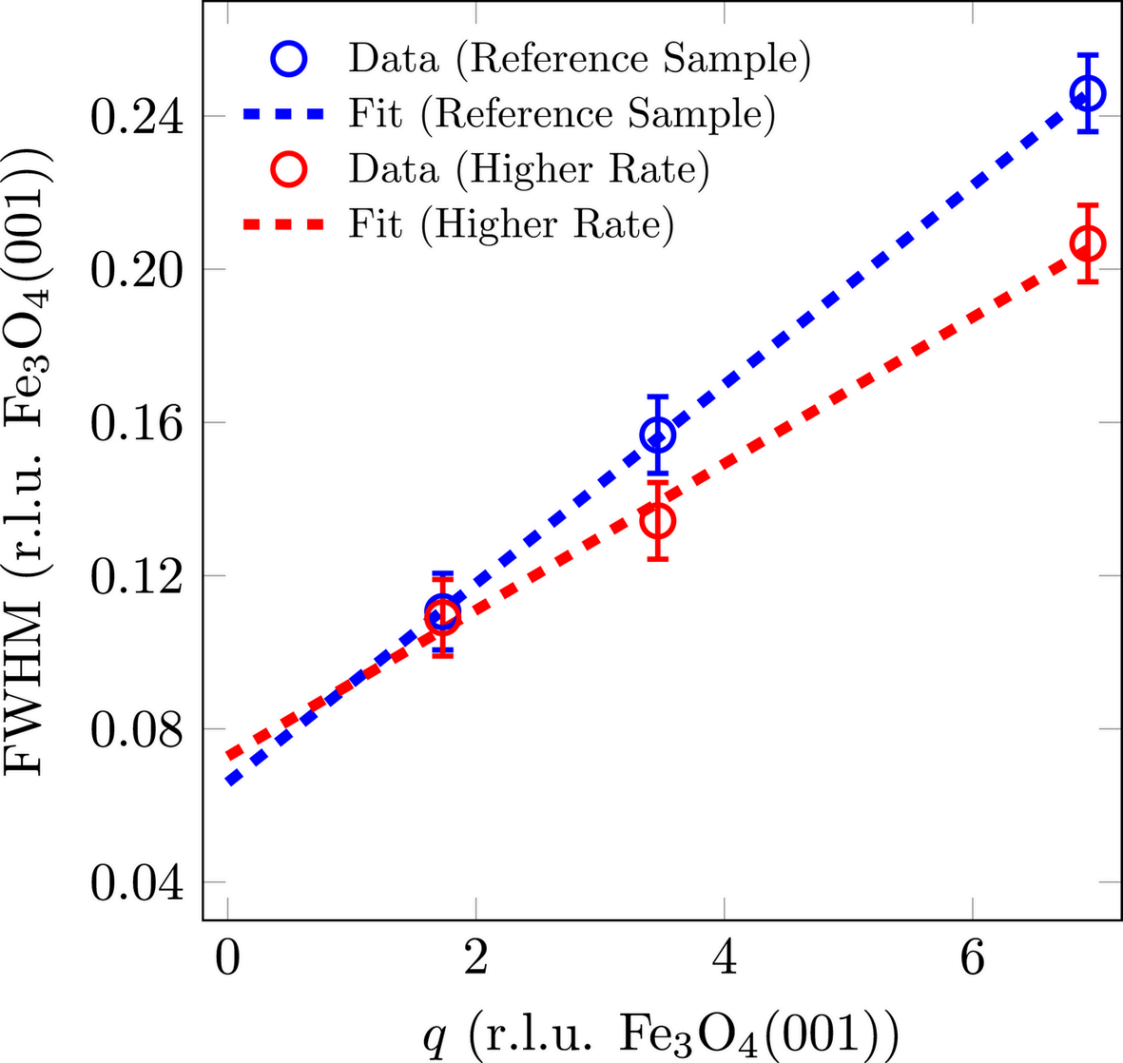
FWHM analysis of (*LLL*) reflections of (001)-oriented Fe_3_O_4_ as a function of the scattering-vector magnitude *q* for different deposition rates. The FWHM of the peaks has been fitted with a linear function.

Fig. 8 ▸ shows the FWHM versus the scattering-vector magnitude *q* for the Fe_3_O_4_(001) reflections. The sample with a higher deposition rate also shows a widening with *q*, which indicates tilting mosaics. For this reason, the FWHM was fitted with a linear function to determine the mosaic size *d*_mosaic_ and the mosaic spread Δσ. Similarly to the crystalline film thickness, the lateral mosaic size with respect to the [111] direction for the sample with a higher deposition rate is slightly lower at *d*_mosaic_ = 10.3 (2) nm than for the reference sample [*d*_mosaic_ = 11.3 (2) nm].

From the slope of the FWHM with *q*, a mosaic spread of Δσ = 1.34 (4)° is obtained for the sample with a higher deposition rate. This is also slightly lower than that for the reference sample [Δσ = 1.82 (4)°], which indicates a sharper distribution of mosaic angles with a higher deposition rate.

### 3.3. Influence of film thickness

The second variation in film growth parameters was a lower film thickness *d*_f_. To achieve this, the deposition time was reduced to one-third of that used for the reference sample while the deposition rate and temperature were kept constant.

Because there was insufficient signal intensity from the magnetite film, no in-plane map could be recorded, while it was possible to record out-of-plane scans.

**Figure 9:**
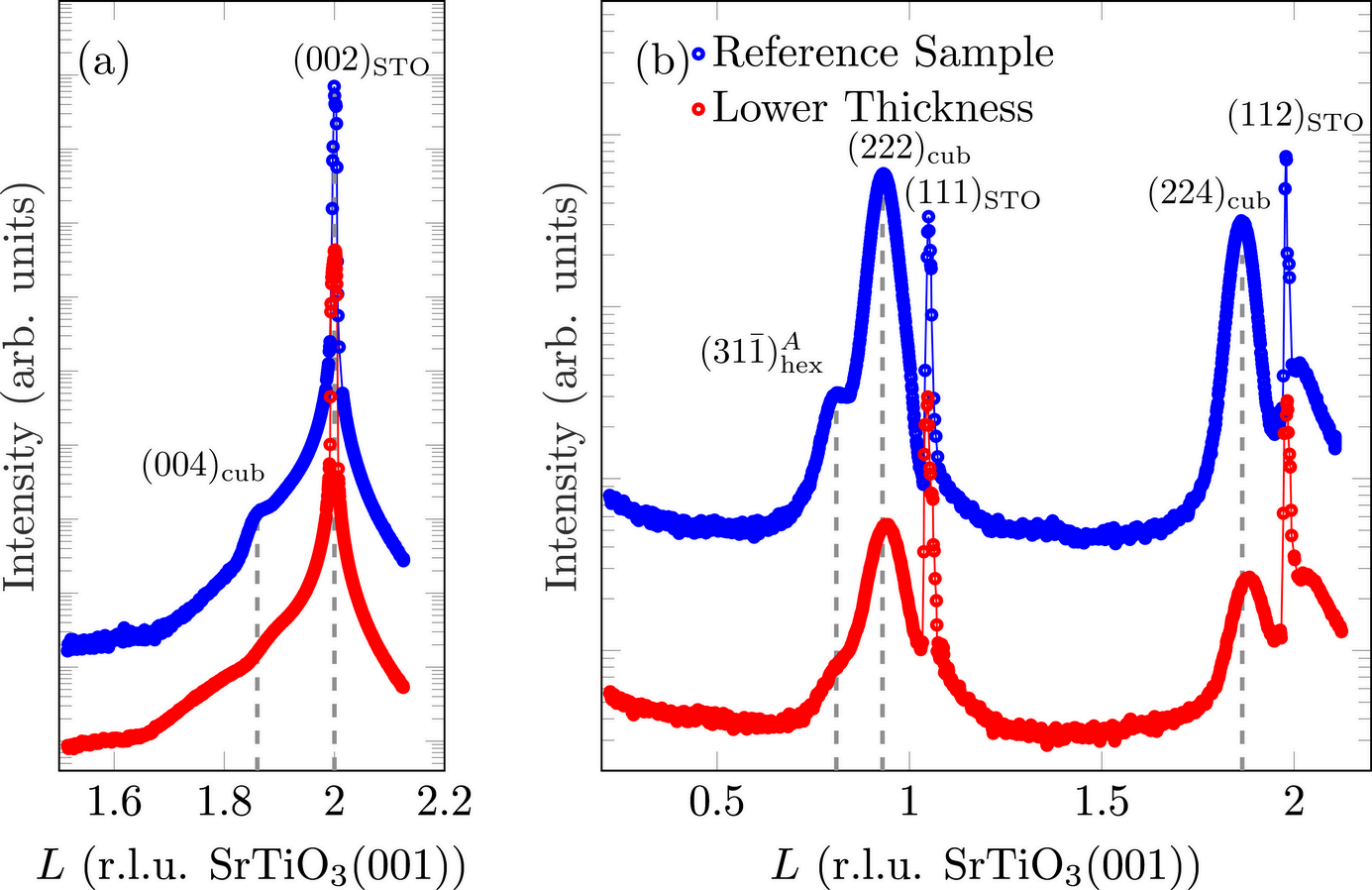
(*a*) Comparison of XRD scans along the (00)-CTR for films of different thicknesses. (*b*) Comparison of *L* scans along the 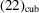-CTR of Fe_3_O_4_(001) for different film thickness. Here, the sharp outliers at *L* = 1 and *L* = 2 are due to diffuse scattering close to very intense substrate Bragg peaks being close to the measured CTRs.

There are no significant qualitative differences either in the XRD measurement along the (00)-CTR or in the *L* scan along the (22)_cub_-CTR (*cf.* Fig. 9 ▸). The intensity of the reflections is evidently weaker in the thinner film, as expected due to the probed film volume. Since (004)_cub_ could not be reasonably fitted due to its low intensity, it was not possible to determine the crystalline layer thickness according to the Scherrer equation.

The intensity ratio between 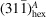 and (222)_cub_ as obtained from the (22)_cub_-CTR of Fe_3_O_4_(001) results in a fraction of (111)-oriented Fe_3_O_4_ of 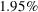 according to equation (2). Compared with the reference sample, this is an increase in the proportion by a factor of 2.5 and corresponds very well to the respective reduction in film thickness. This means that the (111) orientation forms directly at the beginning of film growth and the Fe_3_O_4_ film then continues to grow exclusively in the (001) orientation. The (111) orientation is therefore present as a permanent intermediate layer.

**Figure 10:**
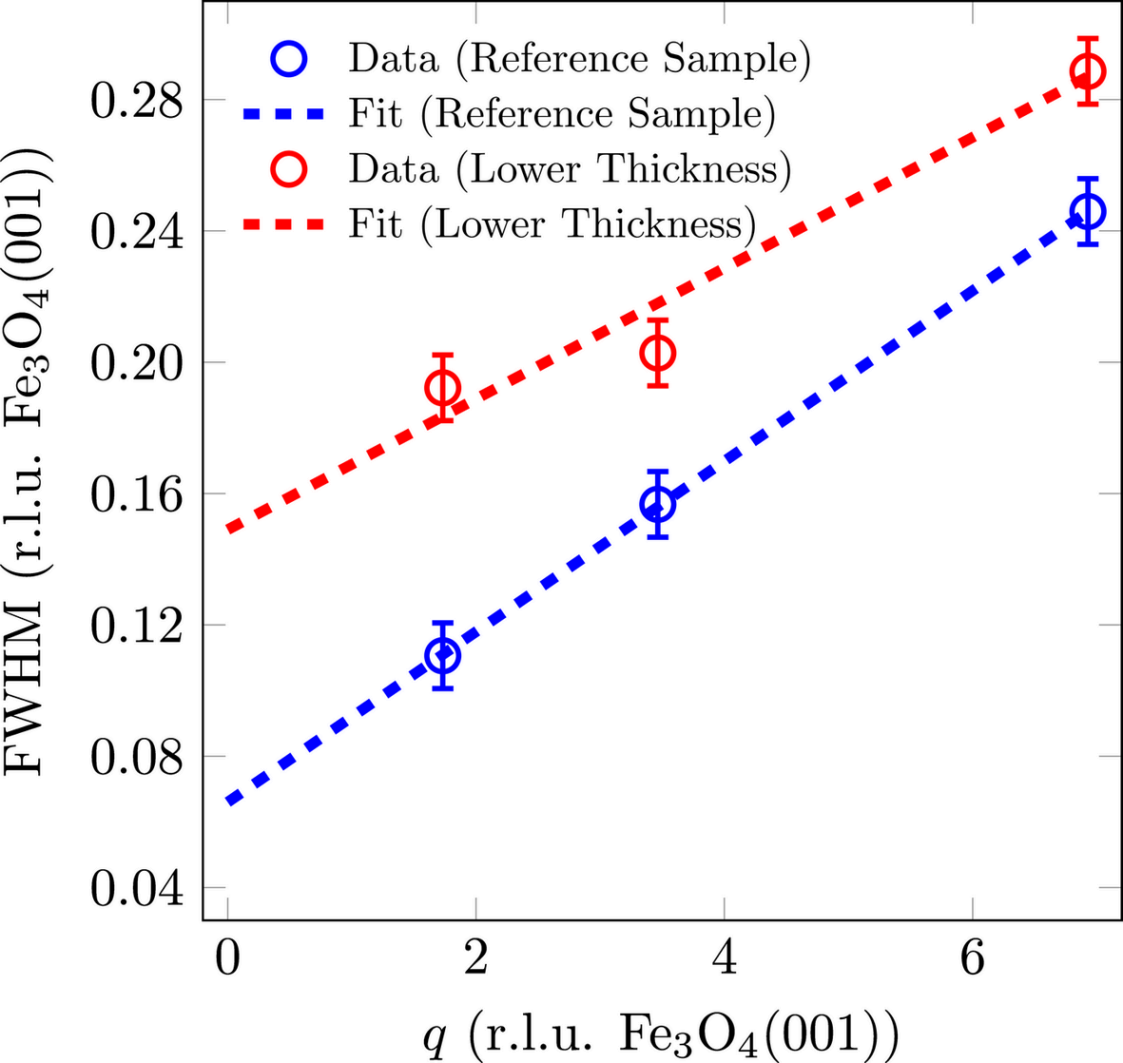
FWHM analysis of (*LLL*) reflections of (001)-oriented Fe_3_O_4_ as a function of the scattering-vector magnitude *q* for different film thicknesses. The FWHM of the peaks has been fitted with a linear function.

The reflections of Fe_3_O_4_(001) also broaden with increasing *q* for the sample with a lower layer thickness (*cf.* Fig. 10 ▸). The fit gives a lateral mosaic size with respect to the [111] direction of *d*_mosaic_ = 5.0 (2) nm and a mosaic spread of Δσ = 1.36 (4)°. Since the crystalline layer thickness *d*_cryst_ could not be determined using the XRD scan along the (00*L*) rod, the projection of the mosaic size perpendicular to the substrate [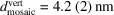] is used to estimate the crystalline film thickness. Accordingly, the crystalline layer thickness *d*_cryst_ must be in-between the vertical mosaic size 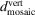 and the evaporated film thickness *d*_f_, *i.e.* 4.0 < *d*_cryst_ < 5.6 nm.

The mosaic spread is lower than for the reference sample and indicates that the distribution of mosaic angles is sharper at the beginning of the growth and broadens with increasing film thickness.

### 3.4. Influence of deposition temperature

The third variation in film growth parameters was a higher deposition temperature. The deposition temperature was increased from 420 to 700°C, while the deposition rate and time (film thickness) were kept constant.

**Figure 11:**
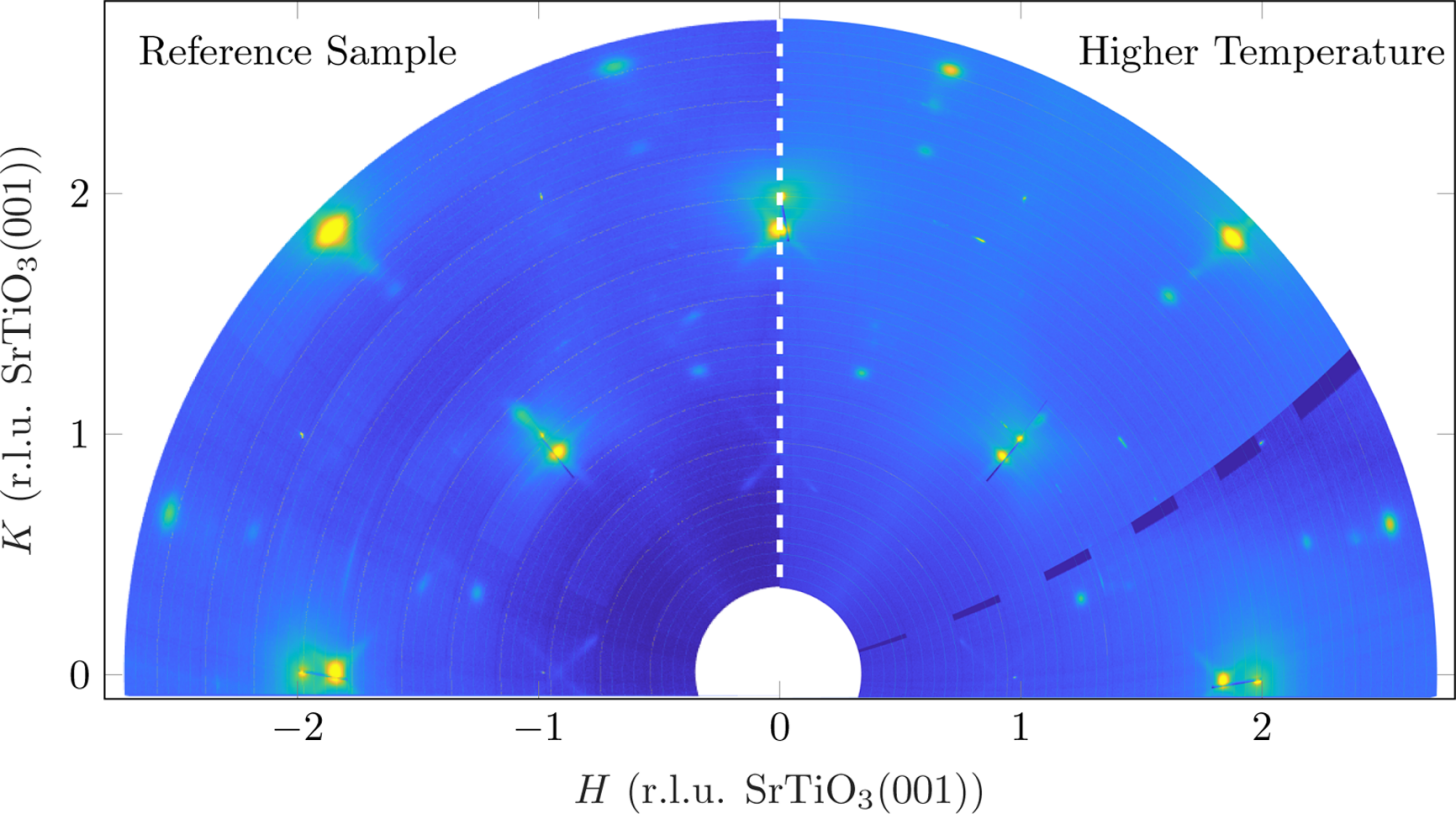
Comparison of the in-plane reciprocal-space maps of the reference sample (left, deposition temperature 420°C) and the sample with increased deposition temperature (right, 700°C). The *L* value is a summation between 0.02 and 0.1 [r.l.u. SrTiO_3_(001)]. The dark regions on the right-hand side are artefacts caused by the overlap of the individual detector images.

Fig. 11 ▸ shows the comparison of the reciprocal in-plane maps for deposition at different temperatures. All reflections of Fe_3_O_4_, from both (001) and (111) orientations, are significantly sharper and more pronounced in the sample that was grown at a higher temperature. This indicates higher crystalline order in the lateral direction for both orientations of Fe_3_O_4_. Furthermore, the cross-shaped weak intensities around the Fe_3_O_4_(001) reflections caused by facets can be seen here. Although these are sharper, they are less pronounced compared with those for the film deposited at lower temperature (reference sample).

**Figure 12:**
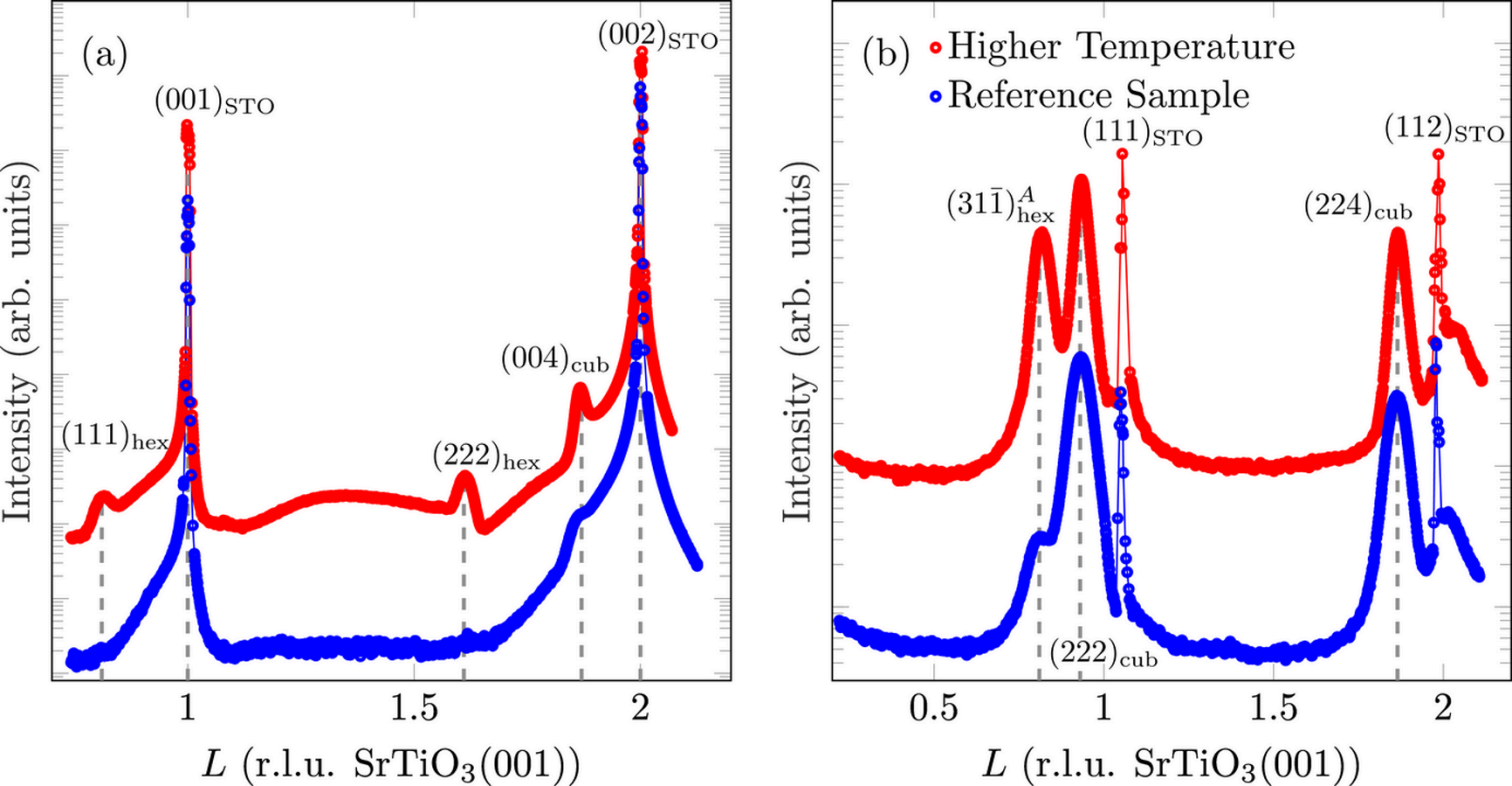
(*a*) Comparison of XRD scans along the (00)-CTR for different deposition temperatures. (*b*) Comparison of *L* scans along the 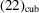-CTR of Fe_3_O_4_(001) for different deposition temperatures. Here, the sharp outliers at *L* = 1 and *L* = 2 are due to diffuse scattering close to very intense substrate Bragg peaks being close to the measured CTRs.

In the XRD scan along the (00*L*) rod [*cf.* Fig. 12 ▸(*a*)], the reflections of the (001) orientation of Fe_3_O_4_ are sharp and clearly pronounced, which indicates a high crystalline order. Furthermore, at *L* = 0.81 and *L* = 1.62, the two reflections of the (111) orientation, (111)_hex_ and (222)_hex_, respectively, are clearly recognisable. Accordingly, Fe_3_O_4_ is present in its (111) orientation in a significant proportion. The *L* scan along the (22)_cub_-CTR of Fe_3_O_4_(001) also provides a comparable result [see Fig. 12 ▸(*b*)]. Here, too, all Fe_3_O_4_ reflections are sharper and more pronounced than for diffraction reflections obtained from the film deposited at lower temperature, especially 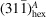 belonging to the (111) orientation. The intensity ratio according to equation (2) results in a proportion of 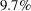 for (111)-orientated Fe_3_O_4_. Compared with the reference sample, this is a significant increase in the proportion of (111) orientation by a factor of 12.6 and confirms previous work that higher temperatures favour the (111) orientation of Fe_3_O_4_ on SrTiO_3_(001) (Takahashi *et al.*, 2012 ▸, 2014 ▸; Leung *et al.*, 2008 ▸).

**Figure 13:**
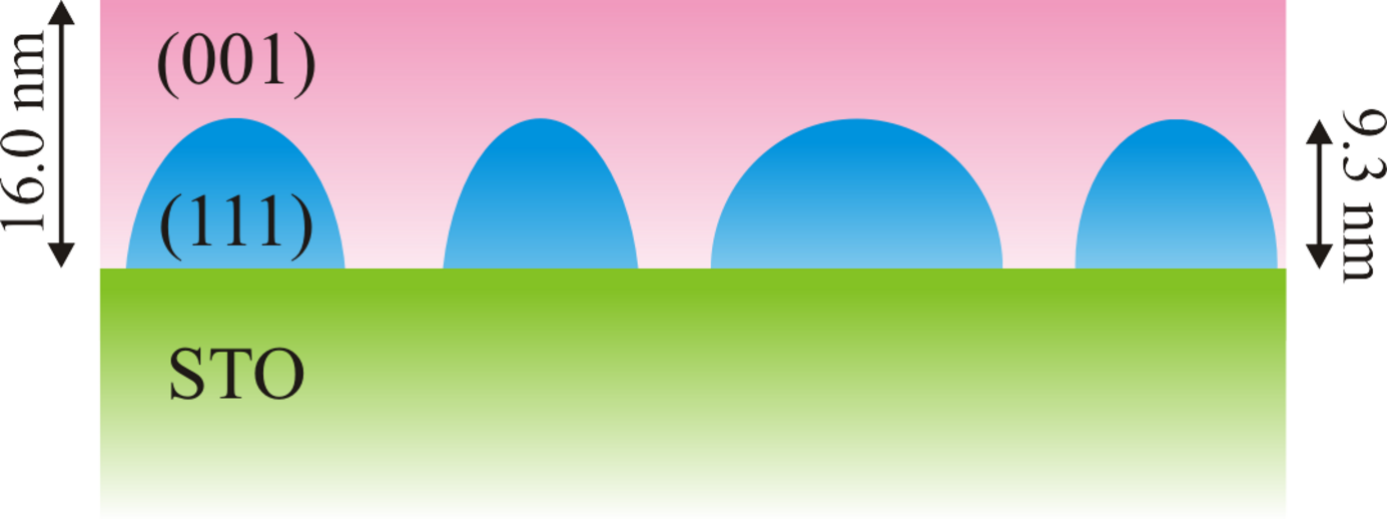
Schematic sketch of the grown film. The initial (111)-oriented Fe_3_O_4_ grows in 3D islands on the SrTiO_3_(001) substrate. Afterwards, the space between these islands is filled and covered by (001)-oriented Fe_3_O_4_. Even though, for reasons of simplicity, the (111) orientation has been sketched here as simple islands on the substrate, it cannot be excluded that an additional thin layer of (111)-oriented Fe_3_O_4_ exists at the interface, meaning that this is a case of layer-plus-island growth (Stranski–Krastanov). This structure applies generally to all samples studied. Only the respective layer thicknesses and the proportion of (111) islands differ.

The FWHM of (004)_cub_ results in a crystalline film thickness of *d*_cryst_ = 16.0 (2) nm, which is in very good agreement with the deposited amount of Fe_3_O_4_ and is the highest value among the samples studied. This is probably due to the increased growth kinetics, which favour a better ordering of the layer. Since reflections of the (111) orientation of Fe_3_O_4_ can be seen in this sample on the (00*L*) rod, these can also be used to determine the crystalline film thickness of the (111) orientation according to Scherrer [equation (1)]. This results in a crystalline film thickness of *d*_cryst_ = 9.3 (2) nm. Since both orientations of Fe_3_O_4_ together far exceed the amount of Fe_3_O_4_ deposited, the (111) orientation cannot be a continuous laminar intermediate layer. Rather, the (111)-oriented Fe_3_O_4_ must be present in the form of 3D islands, covered by the (001)-oriented Fe_3_O_4_ (*cf.* Fig. 13 ▸). In addition, the space between (111)-oriented 3D islands is filled by the (001)-oriented Fe_3_O_4_ film.

A comparable result can also be observed in the other samples. However, since the samples do not show any reflections of the (111) orientation on the (00)-CTR, 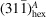 must be used for this analysis. Since additional broadening due to mosaics cannot be excluded, only the minimum crystalline thickness that the (111) orientation must have can be estimated. Nevertheless, the minimum thickness for all samples is significantly higher than what would be expected for a laminar layer structure, *i.e.* yield × deposited thickness, so that the island model is also applicable to the other samples.

**Figure 14:**
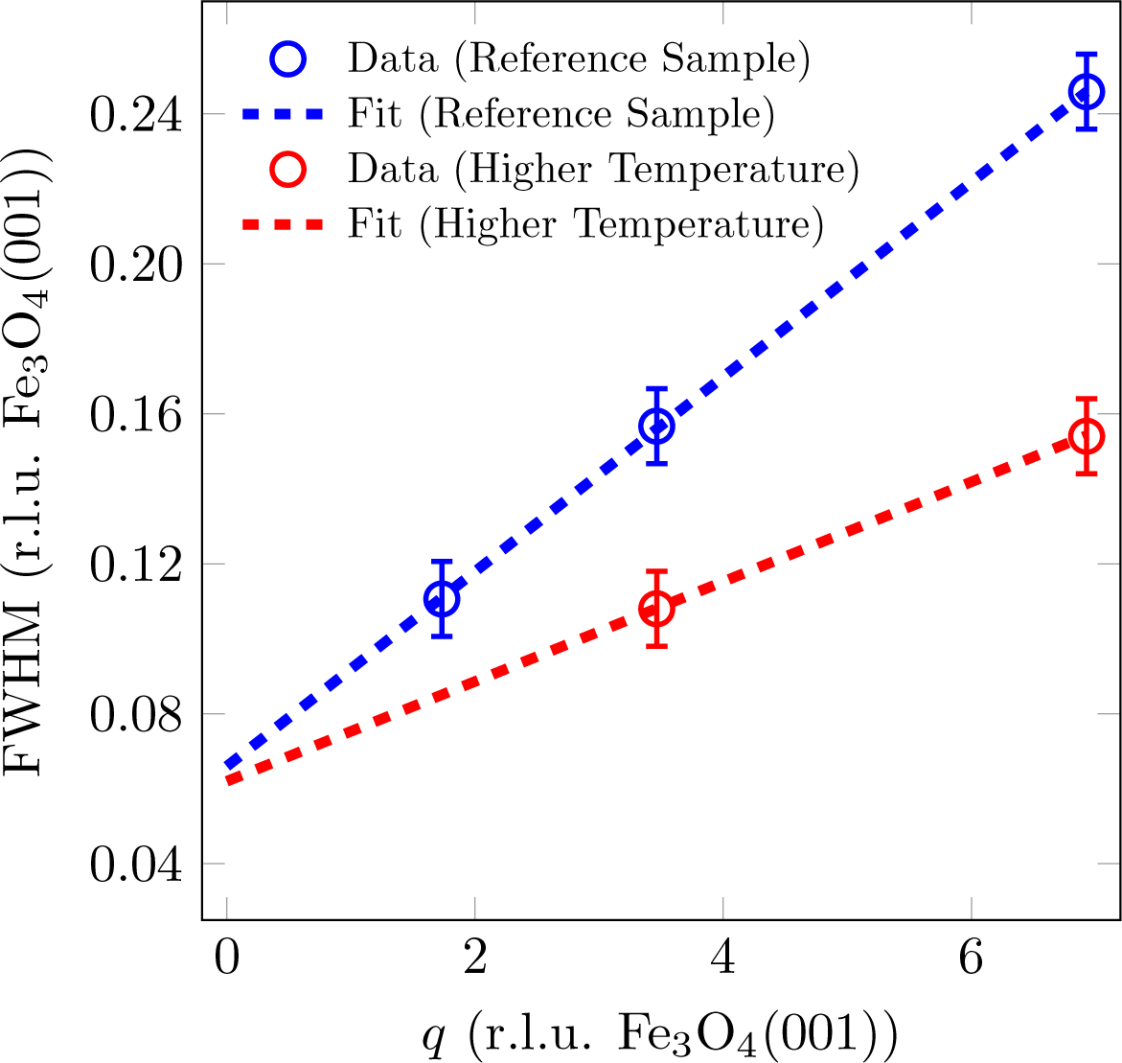
FWHM analysis of (*LLL*) reflections of (001)-oriented Fe_3_O_4_ as a function of the scattering-vector magnitude *q* for different deposition temperatures. The FWHM of the peaks has been fitted with a linear function. As the data point on the (111)_cub_-CTR deviates significantly from the trend of the other data, it was not considered in the fit.

In the same way as with the other samples, there is also a broadening of the Fe_3_O_4_(001) reflections with *q* in the sample that was grown at a higher temperature (*cf.* Fig. 14 ▸). A fit here results in a lateral mosaic size with respect to the [111] direction of *d*_mosaic_ = 12.1 (2) nm and a mosaic spread of Δσ = 0.93 (4)°. The mosaic spread is the lowest of all four samples, which means that the mosaics are more ordered and tend to have a preferred direction, as well as the largest lateral extension.

### 3.5. HEXRD

**Figure 15:**
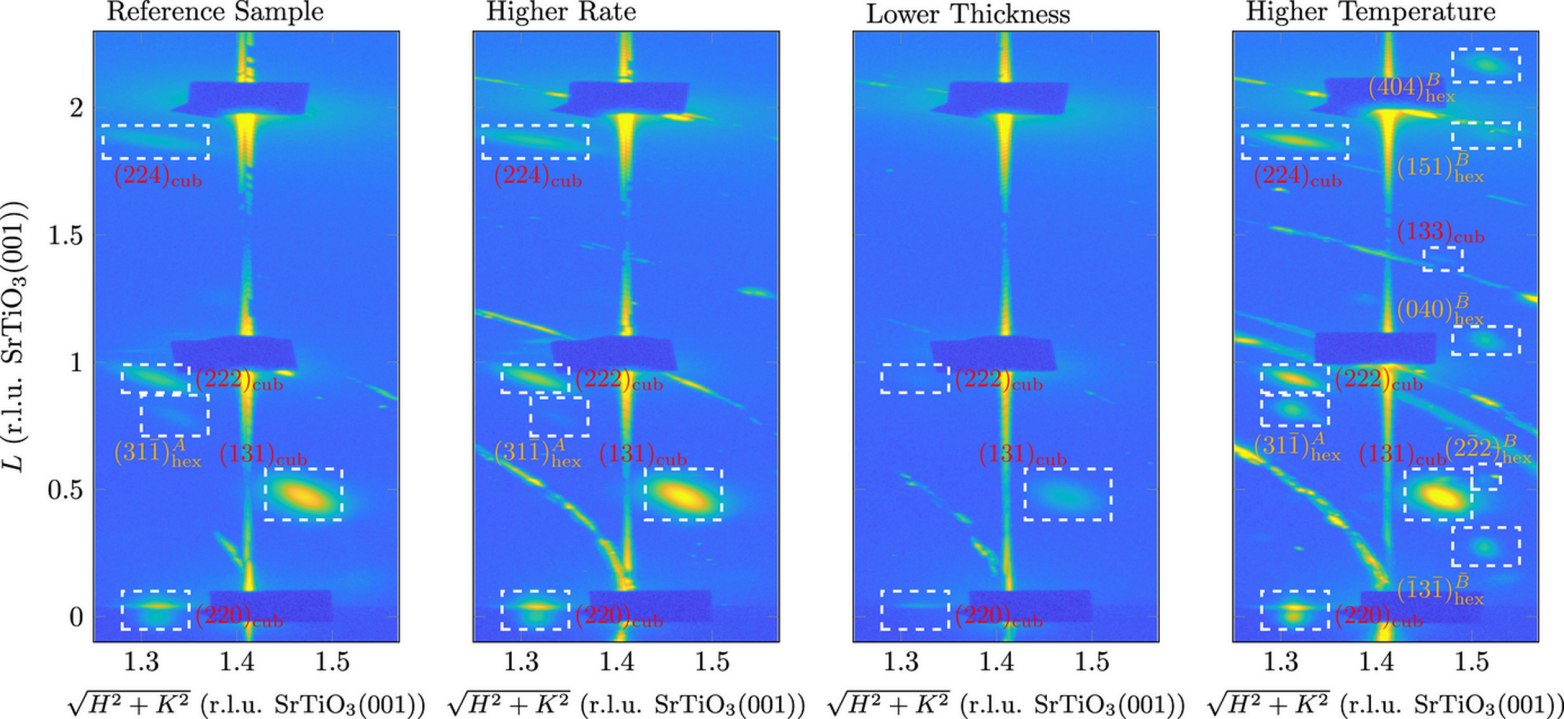
Sections of the recorded HEXRD reciprocal-space map for all four samples. The dark rectangles at the positions of the SrTiO_3_ reflections are caused by beamstops used to protect the detector from the substrate’s bright Bragg reflections.

To gain a deeper insight into the complete in-plane and out-of-plane structure of the ultrathin films, the samples were analysed *a posteriori* using HEXRD. Fig. 15 ▸ shows a section of the reciprocal-space map for the samples analysed near the (22)_cub_- and (11)_STO_-CTRs, where reflections are expected from both the (001) and (111) orientations of Fe_3_O_4_.

As with the GIXRD in-plane map, only the more intense reflections are visible in the thin sample, even from the (001) orientation. In addition to the reflections of the Fe_3_O_4_(001) orientation, 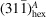 on the (22)_cub_-CTR can be seen in the reference sample and the high-rate sample. In the high-temperature sample, on the other hand, all the expected reflections from the (111) orientation can be seen, except for the one that would be expected at approximately 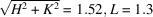 [r.l.u. SrTiO_3_(001)]. However, this is 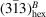, which is significantly weaker than the other reflections due to the low form factor [*cf.* the (133)_cub_ reflection to its left, which is barely visible even at the (001) orientation]. It is noticeable that the (111) orientation in the reference sample and the high-rate sample is slightly laterally distorted or not fully relaxed, as 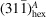 is not fully aligned with the (22)_cub_-CTR, as expected for relaxed Fe_3_O_4_(001), but is slightly shifted towards the substrate reflection. This is not the case with the high-temperature sample, where the film is (almost) completely relaxed.

Analogously to the GIXRD in-plane map (*cf.* Section 3.1), the positions of the Fe_3_O_4_(111) reflections contradict the model proposed by Takahashi *et al.* (2012 ▸). Even though, in the case of the reference sample and the high-rate sample, the (111) orientation is not laterally relaxed, suggesting an initial adaptation to the SrTiO_3_(001) substrate’s lattice, the lateral lattice vectors of the structure proposed by the Takahashi model would result in an expansion of 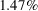 in the 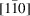 direction and a compression of 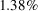 in the [410] direction. Due to the projection of all lateral scattering vector components *q*_||_ over a larger angle range of ±90°, this would be apparent in the HEXRD out-of-plane reciprocal-space map as a double reflection at the position of 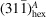, which is not the case in the present measurements.

**Figure 16:**
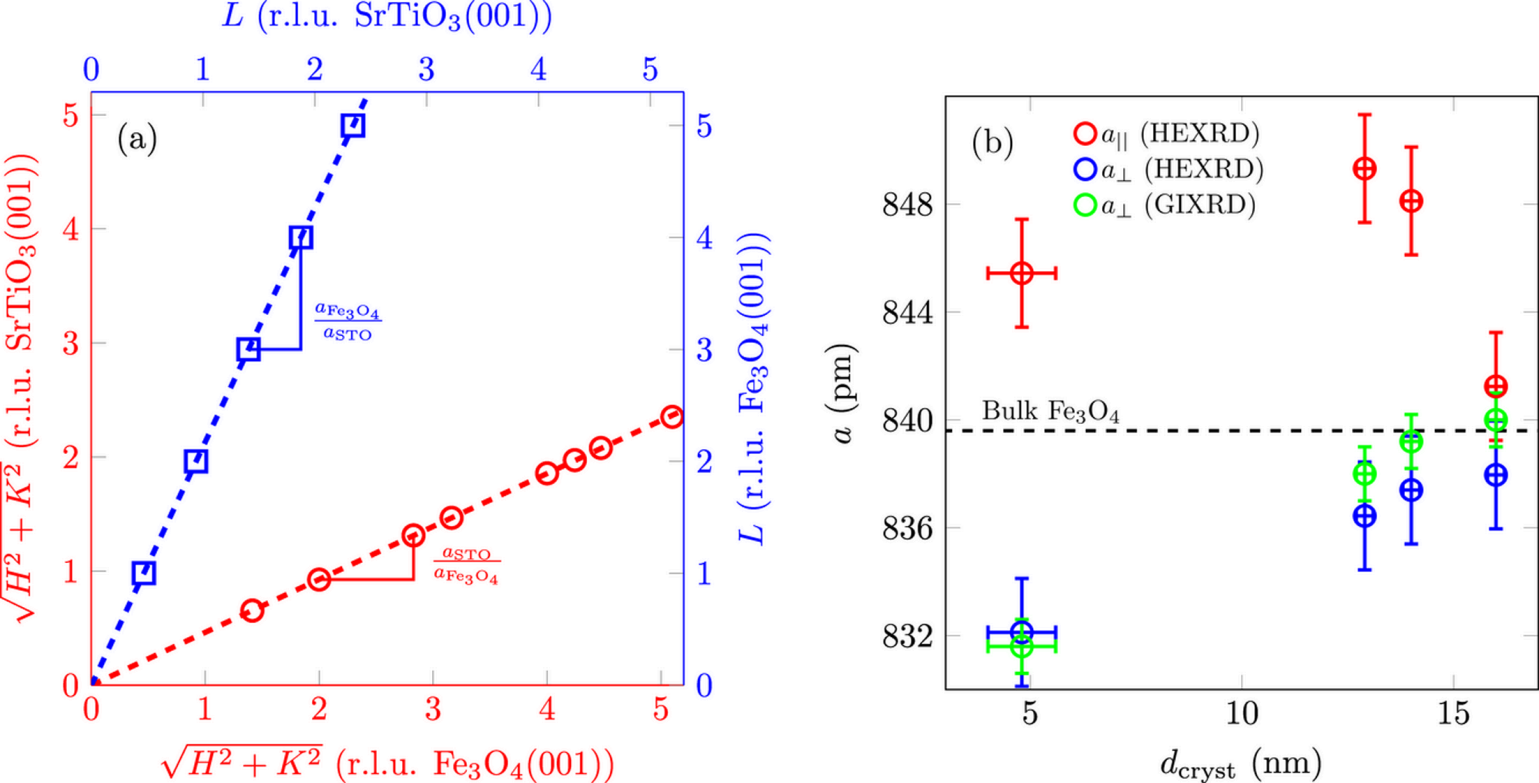
(*a*) Determination of the vertical (blue) and lateral (red) lattice constants for the high-temperature sample. By plotting the fitted reflection positions against their nominal positions, the ratio of the lattice constants of the film and substrate can be obtained from the slope (dashed line). (*b*) Lateral and vertical lattice constants *a*_||_ and *a*_⊥_ versus the crystalline layer thickness *d*_cryst_ of Fe_3_O_4_(001) determined from the fit in the HEXRD measurements. For comparison, the vertical lattice constants *a*_⊥_ determined from the difference of the reflections on the (22)_cub_-CTR are also plotted. The black dashed line denotes the value for bulk Fe_3_O_4_.

To obtain information about the vertical and lateral lattice constants *a*_⊥_ and *a*_||_ for the possibly tetragonally distorted Fe_3_O_4_(001) film, the positions in reciprocal space of the visible reflections of the film were determined. Up to 15 Bragg peaks per sample were fitted with 2D Gaussians to obtain both their out-of-plane and their in-plane positions. If these positions in reciprocal lattice units of SrTiO_3_ are then plotted against their nominal positions in lattice units of Fe_3_O_4_ and fitted with a linear function, the slope of this function corresponds to the ratio of the lattice constants between the substrate and the film [*cf.* Fig. 16 ▸(*a*)].

Fig. 16 ▸(*b*) shows the vertical and lateral lattice constants determined in the above method for the (001) orientation of Fe_3_O_4_, as well as the vertical lattice constant determined from the difference between the (222)_cub_ and (224)_cub_ reflections (*cf.* Section 3.1). The values for the vertical lattice constants determined using different methods agree very well within the range of errors, confirming the validity of both approaches. Within the limits of accuracy, all films except the thickest (high-temperature sample) are tetragonally distorted because their in-plane and out-of-plane lattice constants are different. For the vertical lattice constant, it can be seen that this increases with the crystalline film thickness *d*_cryst_ and approaches the value of bulk Fe_3_O_4_. This means that vertical compression is initially present and the film gradually relaxes as the thickness increases. Note that (except for the reference sample and the low-thickness sample) other parameters (rate, temperature) were also changed for each film thickness. For *d*_cryst_ > 11.9 (2) nm, the Fe_3_O_4_(001) film is fully relaxed in its vertical direction.

In the lateral direction, however, the film is initially expanded and relaxes (excluding the lower-thickness sample) as the film thickness increases, so that it is completely relaxed in the lateral direction as well for *d*_cryst_ > 14.0 (2) nm. How­ever, since the doubled lattice constant of SrTiO_3_(001) (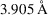) is 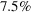 smaller than that of Fe_3_O_4_(001), lateral compression and vertical expansion are to be expected for pseudomorphic growth of ultrathin films and taking into account the theory of elastic strain. Nevertheless, this unusual strain with lateral expansion and vertical compression has already been observed in previous studies on thin films of Fe_3_O_4_, NiFe_2_O_4_ and CoFe_2_O_4_ on SrTiO_3_(001), but so far there is no explanation for this behaviour (Gao *et al.*, 2009 ▸; Hoppe *et al.*, 2015 ▸; Moyer *et al.*, 2012 ▸; Thien *et al.*, 2020 ▸; Rodewald *et al.*, 2020 ▸; Kuschel *et al.*, 2017 ▸).

**Figure 17:**
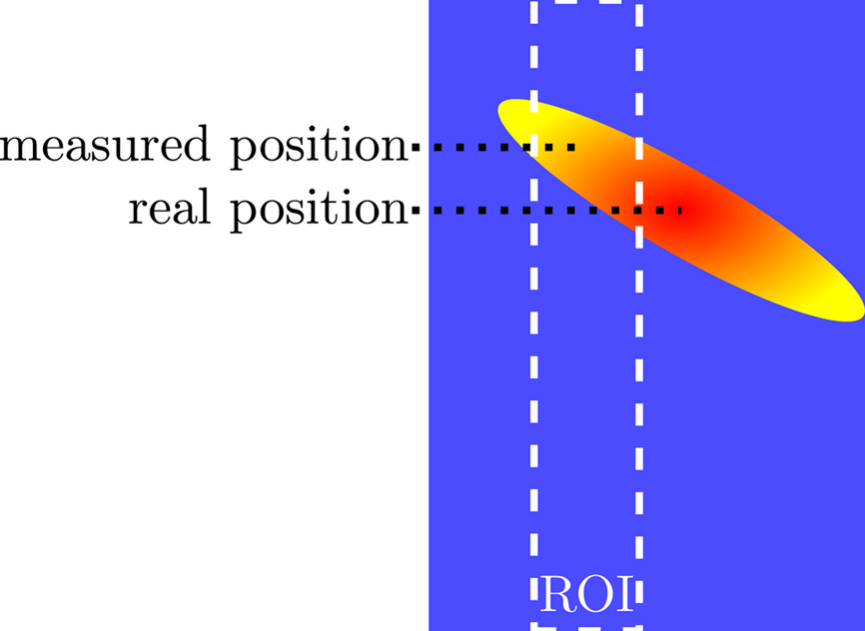
Schematic representation of how the position of a reflection located slightly outside the region of interest (ROI) is misinterpreted.

To determine the vertical and lateral layer distances *c*_⊥_ and *c*_||_ of the (111) orientation, 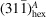 was used, as it was the only reflection that could be fitted for all samples except for that with lower thickness (*cf.* Table 1 ▸). Here, the vertical and lateral layer distances *c*_⊥_ and *c*_||_ denote the layer distance of the (111) orientation of Fe_3_O_4_ in the [111] and 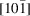 directions, respectively. For bulk Fe_3_O_4_, *c*_⊥_ = 484.7 pm and *c*_||_ = 296.8 pm (Cornell & Schwertmann, 2003 ▸). Besides the high-temperature sample, the vertical layer distances determined from the HEXRD measurements and from the reflection position on the (22)_cub_-CTR (GIXRD experiment) differ significantly from each other. This could be because, contrary to the assumption made in the GIXRD reciprocal-space maps (*cf.* Fig. 2 ▸), the (001) and (111) orientations fully overlap; in the reference sample and the high-rate sample 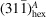 does not coincide exactly with the (22)_cub_-CTR (*cf.* Fig. 15 ▸). Due to its tilted orientation, its position in the *L* scan for the GIXRD experiment is overestimated (*cf.* Fig. 17 ▸).

In all three cases, the (111)-oriented films are highly strained compared with bulk Fe_3_O_4_ (*c*_⊥_ = 484.7 pm and *c*_||_ = 296.8 pm). However, while the reference sample and high-rate sample are vertically expanded and laterally compressed, the high-temperature sample exhibits vertical compression and lateral expansion.

## 4. Summary

Ultrathin Fe_3_O_4_ films were grown on SrTiO_3_(001) substrates under various growth conditions to investigate the influence of these parameters on the formation of the (111) orientation of Fe_3_O_4_. These films were then studied using *in situ* GIXRD and *ex situ* HEXRD, and both in-plane and out-of-plane reciprocal-space maps were recorded to obtain detailed structural information in both the lateral and vertical directions.

**Figure 18:**
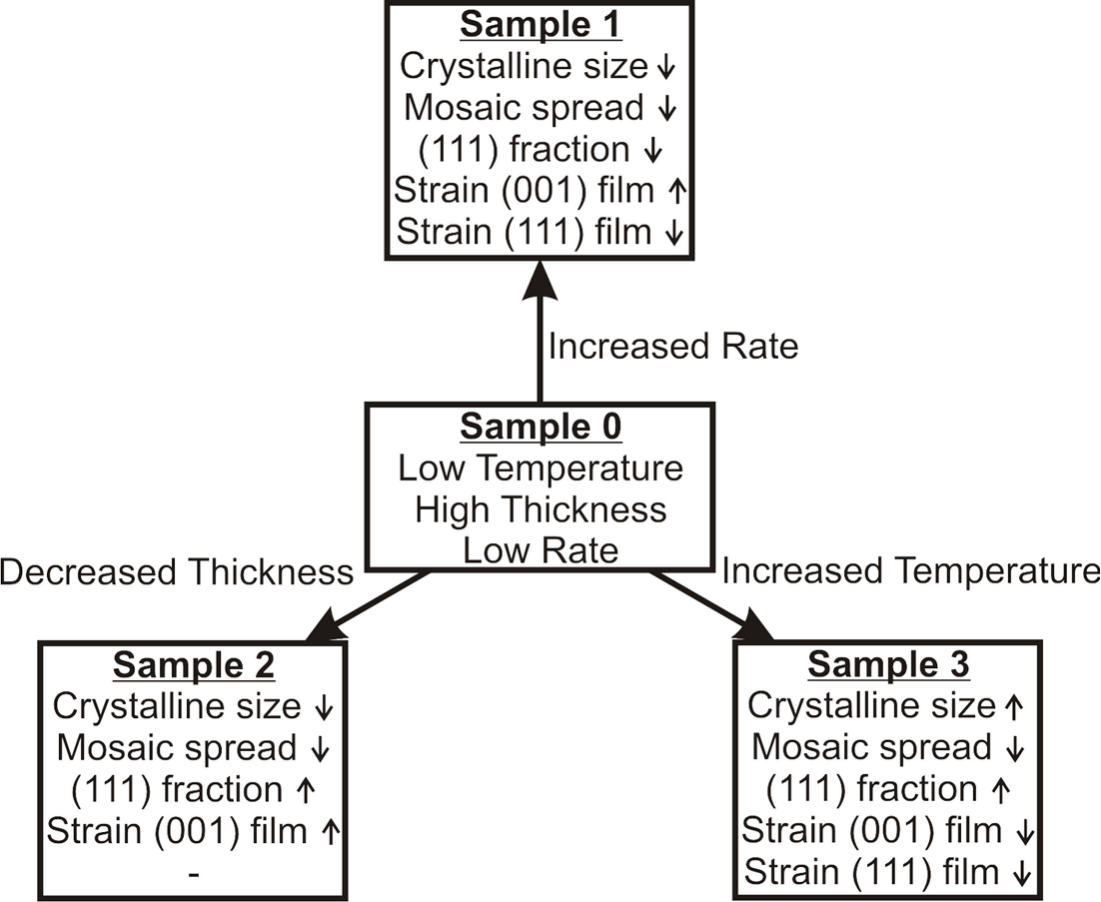
Schematic summary of the main structural changes in the samples studied by changing the growth parameters.

All samples grown exhibit high crystallinity, with the growth conditions and thus the kinetic parameters being crucial for the resulting structure. The lattice constants determined show a dependence on *d*_cryst_ (and other variations of the parameters for film preparation) and an atypical strain that cannot be explained by the standard theory of epitaxy. Moreover, all samples show mosaicity, which indicates misfit dislocations due to the high lattice mismatch between the film and substrate. The main structural changes are summarized in Fig. 18 ▸.

In addition to the expected (001) orientation of Fe_3_O_4_, the (111) orientation is present in all samples studied, forming four rotational domains. The formation of the (111) orientation is favoured by low deposition rates and, in particular, increased deposition temperatures. Investigations on the low-thickness sample show that the (111)-oriented film is predominantly formed at the beginning of film growth and then persists and coexists with the later formed (001) orientation. This (111) orientation forms 3D islands that are subsequently covered or filled by Fe_3_O_4_ with (001) orientation, which confirms previous investigations (Alexander *et al.*, 2025 ▸).

## Figures and Tables

**Table 1:** Lateral and vertical layer distances *c*_||_ and *c*_⊥_ of the (111) orientation of Fe_3_O_4_ determined from the fit in the HEXRD measurements For comparison, the vertical layer distances *c*_⊥_ determined from the measurement of the (22)_cub_-CTR are also plotted. The layer distances of the low-thickness sample could not be determined due to the absence of the 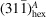 reflection.

	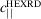 (pm)	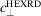 (pm)	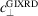 (pm)
Reference sample	294.2 (7)	499.5 (12)	488.0 (5)
Higher rate	295.9 (7)	495.8 (12)	485.0 (5)
Higher temperature	297.8 (7)	480.4 (12)	481.0 (5)
